# In Situ Monitoring of the Effect of Ultrasound on the Sulfhydryl Groups and Disulfide Bonds of Wheat Gluten

**DOI:** 10.3390/molecules23061376

**Published:** 2018-06-07

**Authors:** Yanyan Zhang, Yinli Li, Suyun Li, Hua Zhang, Haile Ma

**Affiliations:** 1 School of Food and Biological Engineering, Zhengzhou University of Light Industry, 5 Dongfeng Road, Zhengzhou 450002, China; zhangyanyan@zzuli.edu.cn (Y.Z.); lyl201314@163.com (Y.L.); Leesuyun@zzuli.edu.cn (S.L.); 2 Collaborative Innovation Center for Food Production and Safety, 5 Dongfeng Road, Zhengzhou 450002, China; 3 Henan Key Laboratory of Cold Chain Food Quality and Safety Control, 5 Dongfeng Road, Zhengzhou 450002, China; 4 School of Food and Biological Engineering, Jiangsu University, 301 Xuefu Road, Zhenjiang 212013, China

**Keywords:** wheat gluten, ultrasonic treatment, sulfhydryl groups, disulfide bonds, in-situ monitoring

## Abstract

Ultrasound treatment can improve enzymolysis efficiency by changing the amounts of sulfhydryl groups (SH) and disulfide bonds (SS) in protein. This paper proposes an in-situ and real-time monitoring method for SH and SS during ultrasound application processes using a miniature near-infrared (NIR) optical fiber spectrometer and a chemometrics model to determine the endpoint of ultrasonic treatment. The results show that SH and SS contents fluctuated greatly with the extension of ultrasonic time. The optimal spectral intervals for SH content were 869–947, 1207–1284, 1458–1536 and 2205–2274 nm, the optimal spectral intervals of SS content were 933–992, 1388–1446, 2091–2148 and 2217–2274 nm. According to the optimal spectral intervals, the synergy interval partial least squares (Si-PLS) and error back propagation neural network (BP-ANN) for SH, SS contents were established. The BP-ANN model was better than the Si-PLS model. The correlation coefficient of the prediction set (*R_p_*) and the root mean square error of prediction (*RMSEP*) for the BP-ANN model of SH were 0.9113 and 0.38 μmol/g, respectively, the *R_p_*^2^ and residual prediction deviation of SH were 0.8305 and 2.91, respectively. For the BP-ANN model of SS, the *R_p_* and the *RMSEP* were 0.7523 and 6.56 μmol/g, respectively. The *R_p_*^2^ and residual prediction deviation (*RPD*) of SS were 0.8305 and 2.91, respectively. However, the *R_p_*^2^ and *RPD* of SS was 0.5660 and 1.64, respectively. This work demonstrated that the miniature NIR combined with BP-ANN algorithms has high potential for in-situ monitoring of SH during the ultrasonic treatment process, while the spectral prediction model of SS needs to be further developed.

## 1. Introduction

Wheat gluten (WG) is the main component of wheat and is one of main by-products of wheat starch processing. WG is the most important plant protein sources, with a high protein content of 80 g/100 g [1]. However, WG is underutilized because of its low solubility. Over the years several investigations on WG have been geared towards improving its solubility and functional properties so as to expand its use in the food industry. Enzymatic hydrolysis is one of the methods that have been found to improve not only the solubility of WG, but has also been found to release many bioactive compounds.

Ultrasound technology, a novel non-thermal physical processing technology, has many applications in food and related fields. Ultrasound treatment can improve the efficiency of proteolysis [2,3,4,5], because of the high pressures, temperatures and shear forces generated by the ultrasonic wave during the enzymatic hydrolysis process which may break chemical bonds of polysaccharide and protein in cell walls [6]. The breakage may increase the surface hydrophobicity and loosen the protein tissue, thereby facilitating the release of bioactive peptides during enzymatic hydrolysis [7].

In order to investigate the mechanism of action of ultrasound pretreatment in the activation of enzymolysis, the effects of ultrasound pretreatment on the molecular structure, secondary structure and microscopic morphology of wheat germ, zein and rapeseed proteins were studied. Our research team performed studies using infrared spectroscopy, circular dichroism spectrometry, fluorescence spectrum, scanning electron microscope, atomic force microscope and other chemical methods. The main reasons behind the improvement in inhibitory activity against angiotensin I converting enzyme (ACE) of the hydrolysates of the wheat protein after ultrasound pretreatment were the changes in fluorescence intensity, surface hydrophobicity, as well as sulfhydryl (SH) and disulfide bonds (SS) after ultrasound pretreatment of the wheat germ. Sweeping frequency pretreatment of corn gluten meal can remarkably raise the degree of hydrolysis and ACE inhibitory activity of zein hydrolysis by altering the SH and secondary structure of zein and by rupturing the smooth surfaces of proteins [8,9,10]. In order to characterize the relationship between ACE inhibitory activity of hydrolysate and SH, SS, surface hydrophobicity and secondary structure elements changes which affected by ultrasound pretreatment. Stepwise multiple linear regressions were performed to describe the quantitative relationships between the structure of WG and the ACE inhibitory activity of hydrolysates. The results show that SH bonds, α-helix, SS bonds, surface hydrophobicity and random coil were significantly correlated with the ACE inhibitory of hydrolysate; the standard partial regression coefficients were 3.729, −0.676, −0.252, 0.022 and 0.156, respectively [11]. The SH and SS contents were the most important property that was affected by enzymolysis effect of WG. The ultrasound treatment improved the degree of hydrolysis and ACE inhibitory activity by changing the SH and SS contents. Therefore, a successive, rapid and accurate method of monitoring the SH and SS contents of protein that can reveal more accurately the mechanism by which ultrasound promotes enzymatic hydrolysis is urgently needed for use in ultrasonic treatment processes. 

Along with the development of detection technology, miniature NIR spectrometry has been applied in a wide range of fields such as modern scientific experiments, medical science, industry, agriculture, astronomical monitoring, and food safety [12,13,14]. Recently, it has been used in monitoring and prediction for food processing on-line and in-situ combined with an optical probe. The aims of this study were: (1) to study the effects of ultrasound treatment on the SH and SS contents of WG; (2) to develop an in-situ and real-time spectroscopy model to monitor the SH and SS contents of WG using a chemometric method; and (3) to establish an efficient variable selection algorithm to improve the performance of the in-situ spectral model.

## 2. Results and Discussion 

### 2.1. The Effects of Ultrasound Treatment on the SH and SS Content

In our previous work, we found that the SH content of protein plays a major role in the improvement of ACE inhibitory activity of the hydrolysates in ultrasonic-assisted enzymatic hydrolysis, while the SS content has a negative effect.

**Figure 1:**
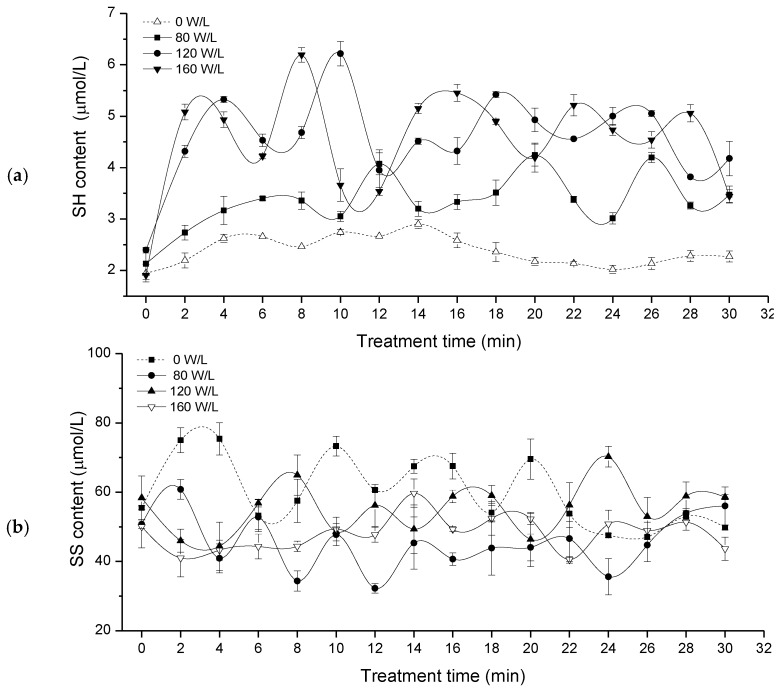
The changes of SH and SS contents during ultrasound pretreatment process. (**a**) SH content; (**b**) SS content.

WG in water is mainly formed by intermolecular SS and intramolecular SS links between gliadin and glutenin molecules. The SH and SS contents represent the degree of tightness of protein spatial structure [15]. The change of SH content and SS content of glutelin in different ultrasonic pretreatment processes (0, 80, 120,160, W/L) were shown in Figure 1.

Figure 1 showed that ultrasound treatment strongly affected the SH content. The greater the power density of the ultrasonic energy was, the more intense was the change. With the prolonged ultrasonic pretreatment time, the SH content fluctuated greatly with wave peak and trough type. With extended ultrasonic time, the SS content fluctuated greatly. This showed that the SH and SS content in the WG suspension is very unstable and affected by the mechanical stirring force. SH content was increased by treatment with ultrasound. On the contrary, treatment with ultrasound generated a lower SS content than that of the control.

Studies have shown that the SH of protein is unstable, susceptible to air, temperature and other effects, and readily oxidized to SS [16]. The accurate and rapid determination of SH and SS content is very important, especially, for in-situ monitoring of SH and SS contents at the chemical reaction site during food processing. Therefore, we focused on in situ spectral methods for monitoring SH and SS contents in the ultrasonic treatment process.

### 2.2. Variable Selection

There exist many collinear variables or variables (starch content, color absorption and temperature fluctuation) irrelevant to the SH and SS in the ultrasonic treatment process. This unwanted information can inevitably weaken the performance of the PLS model. Variable selection is a relevant step in multivariate analysis, since the elimination of non-informative variables leads to better prediction results with simpler calibration models. Thus, it is suggested to calibrate a model by Si-PLS. From Table 1, we can see that, for SH, the optimal number of PLS factors was 10, and the optimal combinations of intervals selected were [1, 5, 8, 17], which corresponded to 869–947, 1207–1284, 1458–1536 and 2205–2274 nm, respectively, in the spectral regions. For SS, the optimal number of PLS factors was 10, and the optimal combinations of intervals selected were [2, 9, 20, 22], which corresponded to 933–992, 1388–1446, 2091–2148 and 2217–2274 nm, respectively, in the spectral regions.

**Figure 2:**
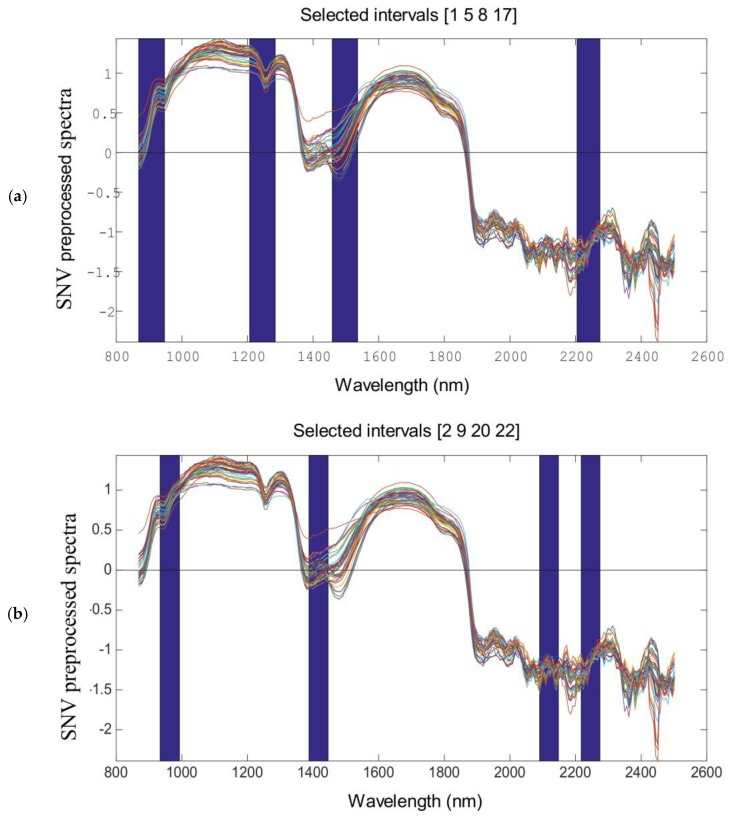
Optimal spectral regions of SH content and SS content selected by Si-PLS (**a**) SH content (**b**) SS content.

The optimal combinations of intervals selected were shown in Figure 2, which shows that these selected spectral regions were correlated with the SH and SS contents of WG in the ultrasound treatment process.

Considering the spectra regions selected by Si-PLS for SH and SS, we found that, the change in SH and SS of WG corresponded to 869–947, 1207–1284, 1388–1536, 2091–2148, 2205–2274 nm. In the NIR region, the characteristic peaks for α-helix were 2056, 2172, 2239, 2289 and 2343 nm, and the characteristic peaks for β-sheet were 2205, 2264 and 2313 nm [17,18]. Thus, the changes in SH and SS contents in the ultrasound treatment process were related to the relative percentage in α-helix and β-sheet.

### 2.3. Development of the Si-PLS Model

**Figure 3:**
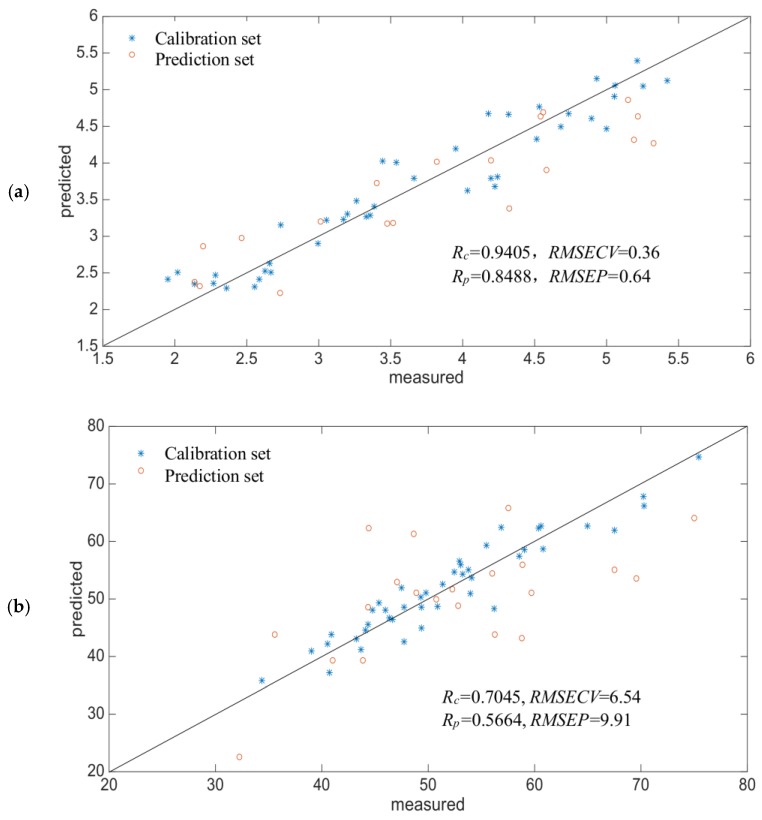
Reference measured versus NIR predicted value of Si-PLS model (**a**) SH content (**b**) SS content.

The Si-PLS model developed for SH content was based on the optimal combinations of intervals, which were from the information for 869–947, 1207–1284, 1458–1536 and 2205–2274 nm. The performance of the optimal model was indicated in boldface in Figure 3a. It showed a scatter plot demonstrating the correlation between reference measurement and the in-situ spectra prediction in the calibration and prediction sets. The value of *RMSECV* was 0.36 µmol/g and *R_c_* was 0.9405 in calibration set. When the performance of the Si-PLS model was evaluated by the samples in the prediction set, the value of *RMSEP* was 0.64 µmol/g and *R_p_* was 0.8488 in the prediction set.

The Si-PLS model developed for SS was based on the optimal combinations of intervals, which were from information for 933–992, 1388–1446, 2091–2148 and 2217–2274 nm. The performance of the optimal model is indicated in boldface in Figure 3b. It shows a scatter plot demonstrating the correlation between the reference measurement and the in-situ spectra prediction in the calibration and prediction sets. The value of *RMSECV* was 6.54 µmol/g and *R_c_* was 0.7045 in calibration set. When the performance of the Si-PLS model was evaluated with the samples in the prediction set, the value of *RMSEP* was 9.91 µmol/g and *R_p_* was 0.5664 in the prediction set.

In general, the Si-PLS model for the SH and SS content in the ultrasonic process was not ideal, and the accuracy needed to be improved.

### 2.4. Development of the BP-ANN Model

**Figure 4:**
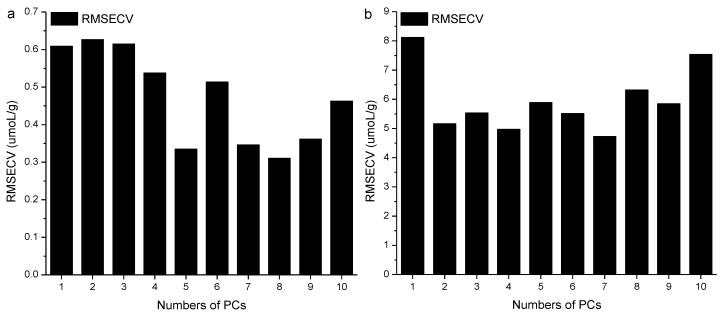
*RMSECV* values of BP-ANN model under different *PCs* of SH and SS contents. (**a**) SH content; (**b**) SS content.

In order to obtain a highly accurate model for SH and SS contents for the in-situ spectra and the chemical values, BP-ANN combined with the optimal spectral regions was used to develop a nonlinear quantitative model. The number of neurons of the input layer was the factor number of the *PC*, which affected the predictive performance of the model. Hence, the numbers of *PC* factors were optimized first. The optimum principal component factor number of the BP-ANN model was then confirmed according to the minimum *RMSECV* value corresponding to the established correction model. Figure 4 shows the relation between the number of *PC* factors and the RMSECV value of the corrected model.

**Figure 5:**
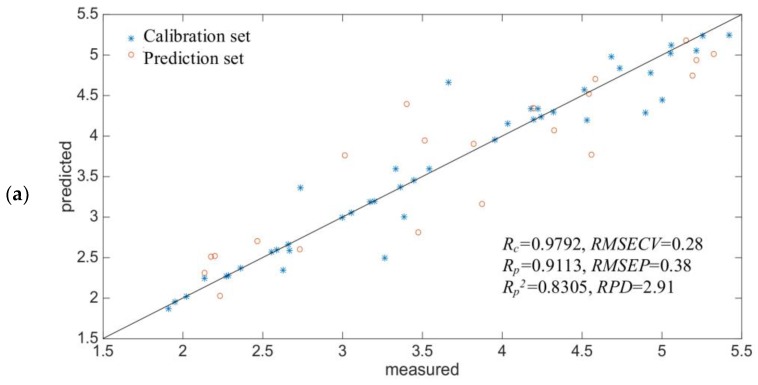

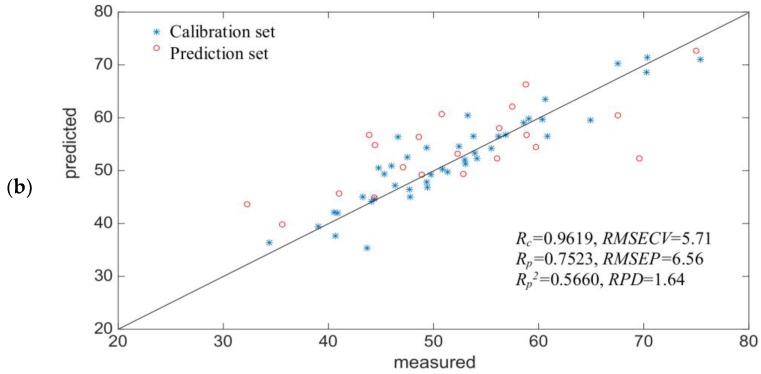
Reference measured versus NIR predicted value of BP-ANN model (**a**) SH content (**b**) SS content.

Figure 4a shows that when the principal fraction was eight, the minimum *RMSECV* of SH was obtained, hence, the number of neurons in the input layer of SH was 8, and the number of neurons was one, which was the measured value of SH. The *R_c_* was 0.9792, and the *RMSECV* was 0.28 µmol/g. The *R_p_* was 0.9113, and the *RMSEP* was 0.38 µmol/g. The correlation between the measured values and the predicted values of the in-situ spectra was shown in Figure 5a.

Figure 4b showed that, when the principal fraction was seven, the minimum *RMSECV* of SS was obtained, so the number of neurons in the input layer of SS was seven, and the number of neurons was one, which was the measured value of SS. The *R_c_* was 0.9619, and the *RMSECV* was 5.71 µmol/g. The *R_p_* was 0.7523, and the *RMSEP* was 6.56 µmol/g. The correlation between the measured values and the predicted values of the in-situ spectra is shown in Figure 5b.

In general, the predictive capability of the BP-ANN model was higher than that of the Si-PLS model. The *R_p_*^2^ of SH was 0.8305, the *RPD* of SH was 2.91. This result indicated that the BP-ANN model has high potential for in-situ predict of SH. However, the *R_p_*^2^ and *RPD* of SS was 0.5660 and 1.64, respectively, indicating this model needs to be further developed.

The SS bond is a covalent bond between sulfur atoms formed by the oxidation of two SH bases. NIR spectroscopy was not very sensitive to SS stretching, which was why the relationship between the SS content and NIR spectrum was more nonlinear.

## 3. Conclusions

In this work, we studied the effects of ultrasound treatment on the SH and SS content of WG. The greater the power density of the ultrasound was, the more intense was the change. With extended ultrasonication time, SH and SS fluctuated greatly. An in-situ and real-time monitoring method for SH and SS contents using a miniature NIR spectrometer, an optical probe, and a chemometrics model was established. The results showed that the optimal spectral intervals for SH content was 869–947, 1207–1284, 1458–1536 and 2205–2274 nm, the optimal spectral intervals for SS content was 933–992, 1388–1446, 2091–2148 and 2217–2274 nm; According to the optimal spectral intervals, the BP-ANN model was better than was the Si-PLS model. Finally, for SH content, the *R_p_* was 0.9113, and the *RMSEP* was 0.38 μmol/g. For SS content, *R_p_* was 0.7523, and the *RMSEP* was 6.56 μmol/g. The *R_p_*^2^ of SH was 0.8305, the *RPD* of SH was 2.91. However, the *R_p_*^2^ and *RPD* of SS was 0.5660 and 1.64, respectively. The BP-ANN model has high potential for prediction of SH, however, the spectral prediction model of SS needs to be further developed. This may be due to the ultrasonic pretreatment process itself, changes in the scope of the content of SS was large and unstable, there may be the measure methods of determination of chemical content of SS value has a certain deviation and the true value for the sample. This work demonstrated that the miniature NIR spectroscopy technique using a fiber optical probe with BP-ANN algorithms has high potential for in-situ monitoring of SH content during the ultrasonic treatment process, the spectral prediction model of SS needs to be further developed.

## 4. Materials and Methods

### 4.1. Materials

WG powder (protein content, 80 g/100 g) was purchased from Xu Zhou OK Wheat Starch Co. (Jiangsu, China). 5,5′-Dithiobis-(2-nitrobenzoic acid) (DTNB), tris(hydroxymethyl) amino-methane, glycine, and carbamide were purchased from Sigma Chemicals Co. Ltd. (St. Louis, MO, USA). All other chemicals and solvents were of analytical grade.

### 4.2. In-Situ Monitoring of the Ultrasonic Treatment Process

**Figure 6:**
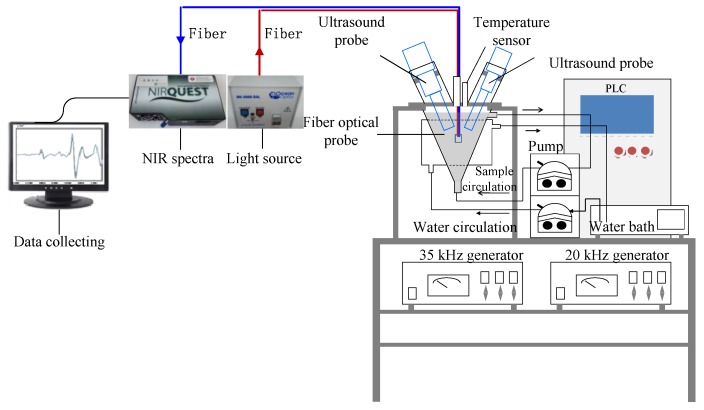
The equipment drawing of in-situ monitoring system in ultrasound treatment process.

The in-situ monitoring system (Figure 6) consisted of four parts: an alternating dual-frequency ultrasound emitter (ADFU), developed by our research team and manufactured by Meibo Biotechnology Co. (Zhenjiang, Jiangsu, China), a miniature NIR fiber optic probe spectroscope (NIRQUEST256-2.5, Ocean Optics, Largo, FL, USA); a portable halogen light source (DH-2000-BAL, Ocean Optics) and a fiber optic probe (TP300, Ocean Optics).

Before ultrasonic treatment, an aliquot of 1.0 L of WG suspension with a substrate concentration of 30 g/L was stirred for 10 min and then placed in the reaction vessel. The two probes were submerged to a depth of 2.0 cm in the suspension, with the two lines inclined to one another at an angle of 60°. The initial temperature of the suspension was 30 ± 2 °C. Ultrasound pretreatment was conducted at frequency of 20/35 kHz in alternate working mode and the duration was 5 s for each treatment was optimized in our previous studies [19]. The NIR instrument was connected to a fiber optic probe with a transmission and reflection module positioned inside the ultrasound reactor below the ultrasound probe and the optical path length was 2 mm (the figure seen our published paper [20]).

This allowed direct contact with the WG suspension during the ultrasound process. The resolution of the miniature near-infrared spectrometer was 6.4 nm and each spectrum was an average of 10 scanning spectra. The NIR spectrometer gathered spectra in the range 850–2500 nm, and we performed measurements every 9.5 nm, resulting in 256 variables.

The sets of experiment were conducted at power densities of 0, 80, 120 and 160 W/L for 30 min. During the ultrasound treatment process, 2 mL samples was taken every 2 min, and then stored at 4 °C for further subsequent analysis. The total number of samples was 64. For each power density condition, NIR spectra were collected in triplicate from three experiments every 2 min. The background spectra of the empty cell were also obtained before each NIR measurement using distilled water at 30 °C. We obtained three spectra for every sample, and the total number of spectra was 192.

### 4.3. Determination of SH and SS Content by Off-Line Methods

The amounts of SH and SS of untreated (control) and ultrasound-treated WG were determined using Ellman’s reagent (DTNB) according to the method of Ellman [21]. Analysis was directly in accordance with the method of Jin et al. [22].

### 4.4. Spectral Data Preprocessing

**Figure 7:**
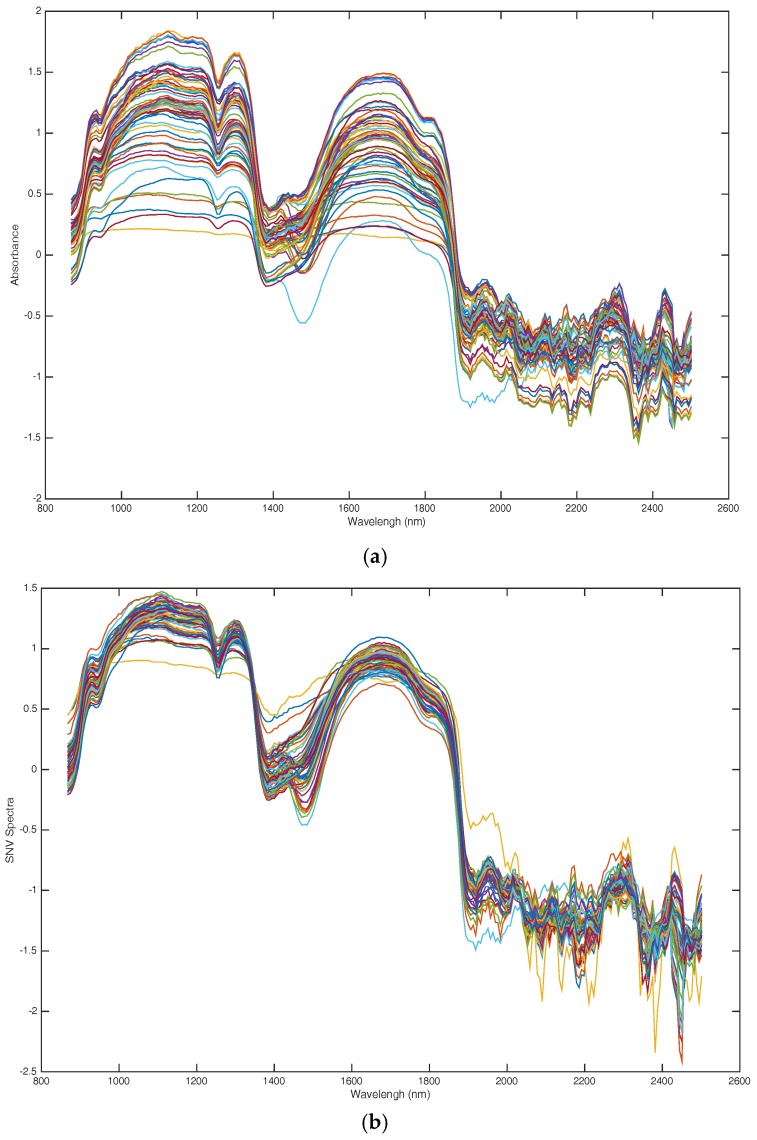
NIR spectra of WG during ultrasound pretreatment process. (**a**) Raw spectra; (**b**) SNV preprocessed.

The spectra acquired during the ultrasonic treatment process also contain information on reflection interference by dissolved particles, baseline drift, machine noise and instrumental noise, which were not related to the changes in protein structure. In order to obtain a more accurate model, the spectra needed preprocessing before the model calibration stage. In this paper, four preprocessing methods, first and second derivative, standard normal variate (SNV) and multiple scattering correction (MSC) were used comparatively in this study. The best preprocessing method was in accordance with partial least square models [23,24,25]. Finally, we find that the modeling effect of SNV was superior to other methods in this work (this result is presented in the Supplementary Data). Raw spectra and preprocessed spectra are presented in Figure 7a, where the SNV spectra are presented in Figure 7b.

### 4.5. Multivariate Analysis

Multivariate analysis methods play an important role in predicting process parameters for a biochemical reaction process using the in-situ spectra system [26,27]. In this paper, a linear model synergy interval partial least square (Si-PLS) and a non-linear model artificial neural network (BP-ANN) were used to develop models for predicting the SH and SS content in the ultrasonic treatment process. In order to divide the calibration/prediction set, one sample from every three samples was selected as the sample in the prediction set and the other two samples were entered to the calibration set. Thus, the measured values and in situ spectra of SH and SS in the process of ultrasonic treatment were divided into two parts, the calibration set (43) and the prediction set (21) for the development of multivariate models Table 2.

#### 4.5.1. Si-PLS

The principle of this algorithm is splitting the data set into a number of intervals, with respect to the variables, and calculating all possible PLS model combinations of two, three or four intervals. In this study, the full spectrum (850–2500 nm) of the samples was divided into 15–30 intervals combined with two, three or four subintervals. The optimal combination of intervals and the number of principal components *PCs* were optimized by cross validation and determined according to the lowest root mean square error of cross validation (*RMSECV*) [28]. The performance of the final model was evaluated by using samples in the calibration set and tested by independent samples in the prediction set. The correlation coefficient (*R_c_*), *RMSECV* of the calibration set, correlation coefficient (*R_p_*) and root-mean-square error (*RMSEP*) of the prediction set were used to evaluate the respective models. Generally, good models should have high *R_c_* and *R_p_* values and low *RMSECV* and *RMSEP* values. In addition, the difference between *R_c_* and *R_p_* or between *RMSECV* and *RMSEP* should be small.

#### 4.5.2. BP-ANN

BP-ANN has excellent nonlinear mapping capability, and has a prominent advantage in dealing with nonlinear relationships. It is the most commonly used nonlinear quantitative method for NIR spectroscopy [25]. Here, the ANN model of SH and SS content were established on the basis of the optimal intervals obtained as described in Section 2.4. The three-layer BP-ANN structure comprises an input layer, a hidden layer, and an output layer. In order to establish a concise and effective model, the date for optimal intervals needed to be reduced in terms of dimensionality, compressed, and then extracted by principal component analysis [29]. According to the *RMSECV*, *R_c_*, *RMSEP* and *R_p_*, the best *PC* factor was selected as the input vector of the BP-ANN model. Finally, the *RMSECV*, *R_c_*, *RMSEP*, *R_p_*, *R_p_*^2^ and residual prediction deviation (*RPD*) value were used to evaluate the precision of BP-ANN prediction model.

### 4.6. Statistical Analysis

All data processing and analysis were conducted were using Matlab 2009b (Mathworks, Natick, MA, USA) for Windows 7. The Si-PLS and BP-ANN algorithm in this work was developed by Chen et al. [28], and the Si-PLS and BP-ANN Matlab codes were downloaded at http://www.models.kvl.dk/.

## Figures and Tables

**Table 1:** Results of selected optimal spectral subintervals for prediction of SH and SS in in the ultrasound treatment process.

Parameters	Number of Subintervals	Selected Subintervals	*PC_s_*	*R_c_*	*RMSEC*	*R_p_*	*RMSEP*
SH (μmol/g)	15	[4, 5, 6, 14]	10	0.9302	0.39	0.7930	0.76
	16	[4, 5, 13, 15]	9	0.9325	0.38	0.7162	0.94
	17	[1, 4, 7]	4	0.9226	0.40	0.8514	0.58
	18	[1, 4, 11]	5	0.9200	0.41	0.8662	0.57
	19	[1, 3, 5]	5	0.9192	0.41	0.8188	0.63
	20	[1, 5, 8, 17]	10	0.9405	0.36	0.8488	0.64
	21	[4, 5, 13]	6	0.9348	0.37	0.8930	0.53
	22	[4, 9, 19]	10	0.9334	0.38	0.7773	0.91
	23	[5, 7, 18, 21]	10	0.9381	0.37	0.7410	0.95
	24	[2, 6, 9]	8	0.9344	0.37	0.8021	0.66
	25	[2, 4, 6, 7]	7	0.9331	0.37	0.8441	0.60
	26	[2, 4, 8, 23]	10	0.9512	0.32	0.7423	0.93
	27	[2, 4, 8, 24]	10	0.9512	0.33	0.7423	0.93
	28	[2, 5, 10, 25]	10	0.9366	0.37	0.8122	0.76
	29	[2, 5, 7]	9	0.9380	0.36	0.8126	0.66
	30	[2, 6, 11, 25]	10	0.9447	0.35	0.7615	0.93
SS (μmol/g)	15	[1, 3, 8, 10]	9	0.6298	8.28	0.6236	7.40
	16	[2, 3, 6, 7]	6	0.6087	8.17	0.4910	8.17
	17	[9, 10, 11, 16]	2	0.5667	7.52	0.6756	9.16
	18	[14, 17, 18]	7	0.6153	7.40	0.5898	9.47
	19	[4, 10, 22, 27]	10	0.8056	5.52	0.5266	12.10
	20	[1, 2, 5]	5	0.5571	8.25	0.4175	8.53
	21	[7, 14, 18]	7	0.6481	6.99	0.4884	10.60
	22	[9, 10, 20]	2	0.5833	7.40	0.6731	7.95
	23	[8, 17, 18, 21]	10	0.7238	6.38	0.4840	11.40
	24	[8, 10, 11, 20]	10	0.4842	9.56	0.5399	9.33
	25	[8, 15, 18]	8	0.6665	6.83	0.3248	12.60
	26	[2, 9, 20, 22]	10	0.7045	6.54	0.5664	9.91
	27	[2, 7, 24]	8	0.6952	6.63	0.5358	9.53
	28	[2, 14, 7, 26]	9	0.6505	7.01	0.6660	7.90
	29	[10, 17, 27]	7	0.6683	6.72	0.6123	8.30
	30	[10, 17, 28]	7	0.6489	6.91	0.6186	8.25

**Table 2:** Reference values for each process parameter in the calibration and prediction set.

Parameters	Units	Subsets	S.N. ^a^	Range	Mean	S.D. ^b^
SH	μmol/g	Calibration set	59	1.9508–5.4225	3.6189	1.0573
		Prediction set	29	2.1976–5.3258	3.7203	1.1061
SS	μmol/g	Calibration set	59	34.3516–75.4038	51.9267	9.0196
		Prediction set	29	32.2722–75.0061	52.4415	10.7267

^a^ S.N., sample number. ^b^ S.D., standard deviation.

## Supplementary Materials

The following are available online.
