# Supplementary material for: In Situ Monitoring of the Effect of Ultrasound on the Sulfhydryl Groups and Disulfide Bonds of Wheat Gluten
Source: Molecules. 2018 Jun 7;23(6):1376. doi: 10.3390/molecules23061376 (PMC6100594; doi:10.3390/molecules23061376)
Supplement: Supplementary file 1 [file molecules-23-01376-s001.pdf]

Supplementary data

Tab.1 Best results from different spectral processing method for the PLS models of SH and SS contents

Parameters	Pretreated method	PC_s	R_c	*RMSEC*	R_p	*RMSEP*
SH	SNV	8	0.8882	0.48	0.8392	0.61
（μmol/g）	MSC	8	0.8884	0.49	0.8385	0.61
	1st Der	10	0.8013	0.55	0.7855	0.78
	2nd Der	9	0.8124	0.64	0.7756	0.74
SS	SNV	4	0.6322	7.41	0.5814	9.66
（μmol/g）	MSC	4	0.6312	7.45	0.5634	9.60
	1st Der	5	0.6055	7.65	0.5044	9.89
	2nd Der	5	0.6123	7.44	0.5146	8.78
